# Modulatory role of regulatory T cells in a murine model of severe equine asthma

**DOI:** 10.1186/s12917-017-1037-0

**Published:** 2017-04-28

**Authors:** Claudio Henríquez, Gabriel Morán, Cristian Carrasco, José Sarmiento, Miguel Barría, Hugo Folch, Benjamin Uberti

**Affiliations:** 1 Pharmacology and Morphophysiology Department, Faculty of Veterinary Sciences, Universidad Austral de Chile, Valdivia, Chile; 2 Immunology Department, Faculty of Medicine, Universidad Austral de Chile, Valdivia, Chile; 3 Pathology Department, Regional Hospital of Valdivia, Valdivia, Chile; 4 Physiology Department, Faculty of Medicine, Universidad Austral de Chile, Valdivia, Chile; 5 Veterinary Clinical Sciences Department, Faculty of Veterinary Sciences, Universidad Austral de Chile, Valdivia, Chile

**Keywords:** Equine asthma, Recurrent airway obstruction, Regulatory T cells, Airway inflammation

## Abstract

**Background:**

It is accepted that T regulatory cells (Treg) control different types of immune responses. In connection with this role, we have recently described an important increase in CD4+, CD25^high^, Foxp3+ lymphocytes in the airway system of horses coursing with an exacerbation of severe equine asthma (EA). To explore the potential role of this population in the resolution of EA inflammation, we used a murine experimental model in which airway neutrophilic inflammation, which is similar to that observed in EA, is induced in mice by continual exposure to *Aspergillus fumigatus* contaminated hay. This model has the advantage that in mice we may induce a reduction of the Treg population using low doses of cyclophosphamide (Cy).

**Results:**

The results indicated that the percentage of Treg cells increased with allergen exposure, as in horses; and animals partially depleted of Treg cells by treatment with Cy showed increased airway inflammation, demonstrated by an increased percentage of neutrophils and specific immunoglobulins in bronchoalveolar lavage fluid (BALF). Furthermore, a histopathologic study of animals that were pretreated with Cy before antigenic challenge showed higher cellular infiltration in the lung and deeper remodeling changes in the bronchi, including epithelial and goblet cell hyperplasia as well as airway smooth muscle hypertrophy.

**Conclusion:**

In this murine model of EA, the reduced number and function of Treg induced by low doses of Cy, which directly correlates with increased airway inflammation and lung infiltration, indicates that Treg may play a major role in the regulation and resolution of EA.

## Background

Equine asthma (EA) [1–3] formerly called inflammatory airway disease (mild asthma) and recurrent airway obstruction (severe asthma) is characterized by periods of acute bronchoconstriction, bronchial hyper-responsiveness, pulmonary neutrophilia, decreased lung compliance and an increase in total lung resistance. Acute episodes of exacerbation are commonly triggered by inhalation of pollutants such as *Faenia rectivirgula*, *Thermoactinomyces vulgaris* and *Aspergillus fumigatus* [4, 5]. Resolution of airways inflammation is likely mediated via multiple mechanisms, with Tregs potentially playing a major contributory role.

It is generally accepted that regulatory T cells play an important role in tumor growth [6], control of autoimmunity [7] and allergic-mediated processes [8]. Since the publication of the seminal paper by Shimon Sakaguchi [9] pointing out the role of CD4+, CD25+ as the T lymphocytes responsible for suppression of the immune response, and because of the important contribution of Fontenot [10], who described the role of transcription factor Foxp3 as a second marker for this lymphoid population, CD4+, CD25+, Foxp3+ cells have been the most studied Treg subpopulation, and their relevance has been evaluated in mice and humans in the context of different immune-mediated diseases [11–18]. However, in domestic animals, information about the role of these cells in immune regulation is scarce. In this context, we have recently demonstrated that in horses with severe EA exacerbation after challenge with moldy hay contaminated with *Aspergillus fumigatus*, the number of Treg cells is greatly increased in lymphocytes recovered from BALF [19]. This increase occurs in parallel with airway inflammation, but, currently, we may only speculate about the importance of Treg in the control and resolution of the acute severe EA process. An approach to answer this question may be attempted through the use of a recently described murine EA model that we have set up in our laboratory [20]. The essence of this model is the use of hay contaminated with *Aspergillus fumigatus* as bedding for experimental mice. After being housed in this condition, Rockefeller (RK) mice show an increase in the percentage of neutrophils in BALF, higher levels of specific antibodies against the fungus in the respiratory tract, and histology that demonstrates intense polymorphonuclear infiltration of the airway: these are important features that also appear in EA [1]. Moreover, in our experimental model, the disease is induced in animals over 5 months of age because younger mice are less sensitive or refractory to the induction of airway inflammation; the same age dependence occurs in the equine species, where the disease is usually observed in horses older than 8 years. In the mice of this experimental model, as also occurs in horses with exacerbation of severe EA, airway inflammation is resolved when animals return to a non-contaminated environment. It is worthy of note that the murine strain used here does not have increased individual susceptibility to airway inflammation, as does occur in EA-susceptible horses [21]. Despite this limitation of the experimental model, the description of immunological processes could be applicable to EA-susceptible and non-susceptible individuals. This murine model of severe EA presented us with the opportunity to modulate the Treg population using low doses of cyclophosphamide (Cy), as described earlier by Barbon [22]. In this context, Askenase’s work [23] showed that Cy given at doses that do not suppress the immune response may increase delayed-type hypersensitivity reactions in mice; our laboratory also reported -in 1980- that low doses of Cy abrogate the phenomenon of antigenic competition [24]; at that time, this effect was attributed to the activation of a T suppressor cell. It since became evident that low doses of Cy potentiate the immune response in several animal models of cancer [25–27].

## Methods

### Animals

RK female mice, aged 2 months (young) or 8 months (old), were used in all experiments. All animals were obtained and maintained at the Animal Facility of the Universidad Austral de Chile. During exposure to *A. fumigatus* contaminated hay, the animals were placed in an isolation room with appropriate ventilation and filtering systems. This study was approved by the Bioethics Committee for the Use of Animals in Biomedical Research of the Universidad Austral de Chile.

The effect of exposure to the fungus was evaluated in 4 groups of animals: non-exposed young animals (*n* = 8), exposed young animals (*n* = 8), non-exposed old animals (*n* = 8), exposed old animals (*n* = 8). The measured outcome for fungus exposure was relative Treg content (CD4+, CD25+, Foxp3+) in spleen.

The effect of cyclophosphamide on airway inflammation was evaluated on three groups of 2 month old animals: not exposed to fungus and not treated with cyclophosphamide (*n* = 6), exposed to fungus but not treated with cyclophosphamide (*n* = 6), and treated with cyclophosphamide and exposed to fungus (*n* = 6).

### *Aspergillus fumigatus* culture, protein extraction and hay contamination

Culture of *Aspergillus fumigatus* was carried out in two steps. The fungus was first cultured in peptone agar for 7 days at 37 °C and then in malt broth for 7 additional days. The resulting fungi were used for soluble protein extraction and to contaminate the mouse bedding. For protein extraction, fungi were blended and subsequently sonicated (Ultrasonic Homogenizer, series 4710 Cole Parmer, USA). The resulting suspension was centrifuged at 12.500×g for 10 min, and the supernatant containing soluble proteins was stored at −20 °C for later use. To expose mice to *Aspergillus fumigatus*, hay was sterilized and sprinkled with water containing fungus spores. The contaminated hay was then introduced into plastic bags and incubated for 48 h or more at 37 °C until mold contamination became visible. At this point, the contaminated hay was used as mice bedding. During the experiment, animals were maintained under these conditions according to the design of each study protocol.

### Cyclophosphamide administration and induction of bronchial inflammation

Groups of mice were intraperitoneally treated with Cy at a dose of 20 mg/kg of body weight, for three consecutive days immediately before starting the antigenic challenge. As controls, groups of mice were injected with saline and exposed or not exposed to *Aspergillus fumigatus* contaminated hay. Experimental groups were maintained in this condition for different lengths of time, and were then euthanized with an overdose of pentobarbital at days 5, 11 and 17. In each case, blood samples, BALF, spleen and lung tissue were obtained for analysis.

### Obtention, processing and analysis of samples

Samples of blood, BALF, spleen and lung were obtained 5, 11 and 17 days after the challenge conditions began. Blood (200 μl) was obtained each time from the treated and control mice and allowed to clot; the collected sera were stored at −20 °C until use. BALF was obtained from previously euthanatized mice; for this purpose, the trachea was exposed and cannulated with a 24G Teflon I.V. catheter. BALF was collected with saline solution (3 × 0.5 ml), and cells were centrifuged once at 500 g for 7 min. Pelleted BALF cells were resuspended in PBS and the total number of leucocytes was counted using a Neubauer chamber. A total of 100.000 cells in a cytocentrifuged preparation of BALF stained with May-Grünwald’s – Giemsa were differentiated under light microscopy, according to classical cell morphology. Lung and spleen were obtained after sacrifice with no previous intervention. Lungs were fixed in 4% formalin and processed for histology, while spleen was teased in cold-culture medium, and the cell suspension was filtered through gauze and adjusted to the desired concentration for flow cytometry.

### Flow cytometry analysis

**Fig. 1:**
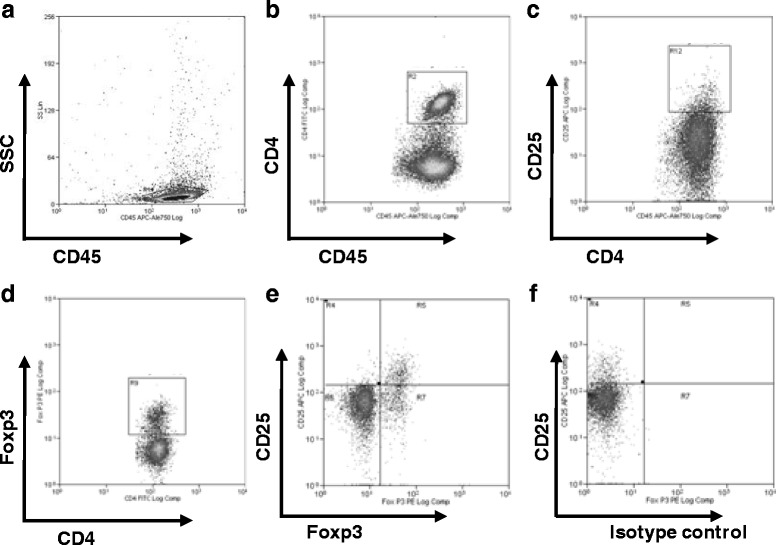
Representative figures of gating strategy for Treg determination in mice spleen. For this purpose a gate was positioned around lymphocytes (**a**), and the CD4+ population was gated (**b**) in order to determinate the percentage of CD4+ cells that express markers CD25 (**c**) and Foxp3 (**d**) within the CD4 subset. Finally, co-expression of CD25 and Foxp3 were measured within CD4 cells (**e**); isotype control for Foxp3 was used as control (**f**)

To determine the impact of exposure to *Aspergillus fumigatus* contaminated hay, age and Cy treatment on the Treg cell population, a suspension of splenocytes adjusted to a concentration of 1 × 10^6^ cells per ml was washed with PBS (pH 7.2) and then resuspended in flow cytometry staining buffer (eBioscience). For the detection of different markers, cells were incubated with the following eBioscience monoclonal antibodies: APC- eFluorTM 780-labeled anti mouse CD45, PE-Cy5-labeled anti mouse CD3 (145-2C11), FITC-labeled anti mouse CD4 (RM4–5), APC-labeled anti mouse CD25 (PC61.5) and PE-labeled anti mouse Foxp3 (FJK16s), according to the manufacturer’s recommendations. At least 30.000 events were assayed using a Beckman Coulter, CyAn™ ADP Analyzer, and data were analyzed using Summit v4.3 software. To determine different cell populations, lymphocytes were gated using SSC and the CD45 marker; then, the CD4 population was visualized and the CD25 positive-cell content was analyzed. Later, Foxp3-positive cells were determined within the total CD4 subpopulation, and the co-expression of CD25 and Foxp3 was determined (Fig. 1).

### Analysis of antibody production

The levels of *Aspergillus*-specific immunoglobulins in BALF and serum were determined by ELISA. Briefly, 96-well polystyrene microplates (Sarstedt, USA) were coated with 10 μg/ml of *Aspergillus fumigatus* total protein that was dissolved in carbonate buffer (pH 9) and incubated overnight. The plates were blocked with a solution of 5% non-fat milk followed by the addition of 100 μl of the appropriate dilution of BALF (1:1) or serum (1:100) into each well. The plates were incubated at 37 °C for 2 h, washed three times and incubated with peroxidase-conjugated anti-mouse IgE, IgG1 or IgG2a antibodies (Santa Cruz, Inc., USA) for an additional 2 h at 37 °C. After incubation, the plates were thoroughly washed, and the reaction was revealed with o-phenylenediamine (OPD; Sigma, USA). The colorimetric reaction was evaluated at 450 nm using a microplate reader (ELx800, Bio-Tek Instruments, USA).

### Histological analysis

For histological sections study, control or experimental lung tissues were fixed in 4% formaldehyde, embedded in Paraplast, cut into 5-μm sections and stained with hematoxylin & eosin.

### Statistical analysis

SigmaPlot 11.0 was used for statistical analyses. Descriptive statistics by group were run on the data, and the Kolmogorov–Smirnov test showed that the data were normally distributed. ANOVA was performed to compare the differences in CD4+, CD25+, Foxp3+ subpopulation percentages, and this was followed by a Tukey test. Overall, *p* values <0.05 were regarded as significant.

## Results

### Splenic Treg cell percentages increase in mice as a function of age and exposition to *Aspergillus fumigatus* contaminated hay

**Fig. 2:**
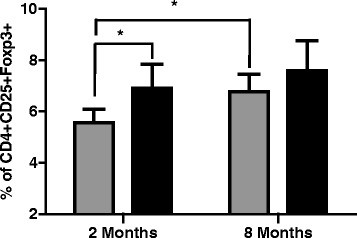
Percentage of CD4, CD25, Foxp3 positive cells in spleen of 2 and 8 month old mice exposed () or non-exposed () to *Aspergillus fumigatus*. Data are presented as Median ± SD. *p* < 0.05 (*n* = 8)

As a first approach, we determined the relative percentage of CD4+, CD25+, Foxp3+ cells in the spleens of two and 8 month old mice by flow cytometry. The results indicated that younger mice had significantly less Treg (5.19% ± 0.45) than older mice (6.77% ± 0.89) prior to Aspergillus exposure, and that the percentage of Treg cells significantly increases only in young mice upon 5 days of exposure to *Aspergillus fumigatus* (from 5.56% ± 0.53 to 6,91% ± 0,94 after mold challenge) (Fig. 2).

### Low doses of cy induce a reduction of splenic Treg in mice exposed to *Aspergillus fumigatus* contaminated hay

**Fig. 3:**
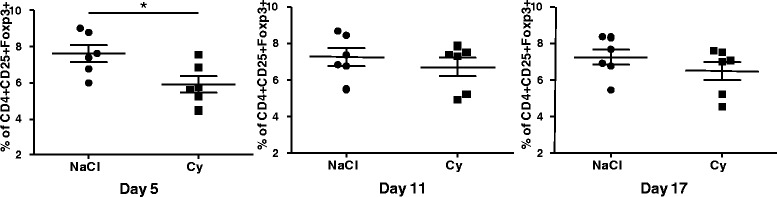
Effect of treatment with low doses of cyclophosphamide on the relative percentage of CD4+, CD25+, Foxp3+ positive cells in the spleens of mice exposed to *Aspergillus fumigatus*, compared to animals injected with the same volume of isotonic saline solution and exposed to the fungus, 5, 11 and 17 days after treatment. *p* < 0.05 (*n* = 6)

Knowing that exposure to *Aspergillus fumigatus* contaminated hay induces an increase of the CD4+, CD25+, Foxp3+ population in mice, as a next step, we tested the effect of low doses of Cy in partially reducing the splenic Treg population, following Barbon et al.’s protocol [22]. In short, before antigenic challenge, the mice were injected daily three times with 20 mg/kg, and the results, which are shown in Fig. 3, indicate that Cy reduced the percentage of CD4+ cells that carried the regulatory markers Foxp3 and CD25. In this experiment, the Treg population was reduced from 7.59% ± 1.17 to 5.90% ± 1.11 5 days after the last dose of the cytostatic drug, indicating that Cy does not induce changes in the percentage of total CD4+ cells compared to the CD3+ population, in total accordance with the results reported previously by Barbon et al. [22].

### The reduction of Treg population -induced by low doses of cy- showed a positive correlation with an increased airway inflammatory response in mice exposed to *Aspergillus fumigatus* contaminated hay

**Fig. 4:**
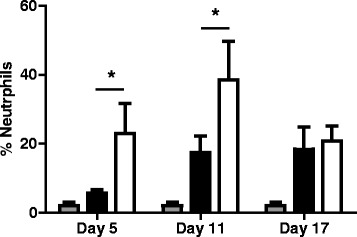
Effect of treatment with low doses of cyclophosphamide on the relative percentage of neutrophils obtained from BALF in non-exposed controls (), compared with animals treated with isotonic saline solution and exposed to *Aspergillus fumigatus* (), and animals that were pretreated with cyclophosphamide and exposed to *Aspergillus fumigatus* (). Data are presented as Median ± SD. *p* < 0.05 (*n* = 8)

Next, to evaluate if the reduction of Treg induced by Cy influenced the inflammatory allergic airway response in mice exposed to *Aspergillus fumigatus* contaminated hay, and knowing that the challenge in this murine experimental model could replicate many clinical findings of horses with acute exacerbation of severe asthma, we evaluated neutrophil percentages in BALF and specific antibodies levels against *Aspergillus fumigatus* in BALF. As observed in Fig. 4, BALF neutrophil percentage was greatly increased in mice pretreated with Cy before exposure to *Aspergillus fumigatus* contaminated hay, compared to animals that were only exposed to the mold. This increase of neutrophils in the Cy-treated group was evident and statistically significant at days 5 and 11 post antigenic challenge. Since the airway inflammatory response to *Aspergillus fumigatus* in horses and in the animals of our murine model is mediated at least in part by the onset of hypersensitivity type I and III, it was interesting to determine specific antibodies against the allergen in BALF obtained from animals challenged with the mold, either treated or not treated with the cytostatic drug.

**Fig. 5:**
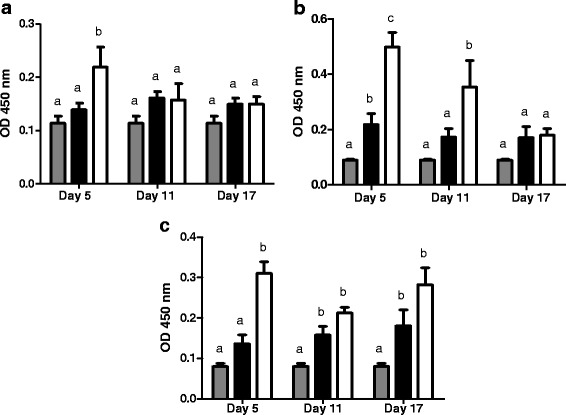
IgE (**a**), IgG1 (**b**) and IgG2a (**c**) levels in control animals (), animals challenged with *Aspergillus fumigatus* and injected with saline () and animals pretreated with cyclophosphamide and challenged with *Aspergillus fumigatus* (). Data are presented as Median ± SD. *p* < 0.05 (*n* = 8)

Figure 5 shows *Aspergillus fumigatus*-specific IgE, IgG1 and IgG2a levels in mice that were not exposed and in mice exposed to contaminated hay and either treated or not treated with Cy. IgE levels were significantly higher in the Cy-treated and *Aspergillus-*exposed group only on day 5 (Fig. 5a). IgG1 levels were significantly different between all three experimental groups on day 5 (i.e. control group, *Aspergillus-*exposed group, and Cy-treated and *Aspergillus-*exposed group); on day 11, IgG1 levels were only significantly higher in the Cy-treated and *Aspergillus-*exposed group; by day 17, there were no significant differences between groups (Fig. 5b). IgG2a levels were significantly higher only in the Cy-treated and *Aspergillus-*exposed group on day 5; on day 11 and 17, IgG2a levels were significantly higher in both *Aspergillus-*exposed groups than in the control group (Fig. 5c).

**Fig. 6:**
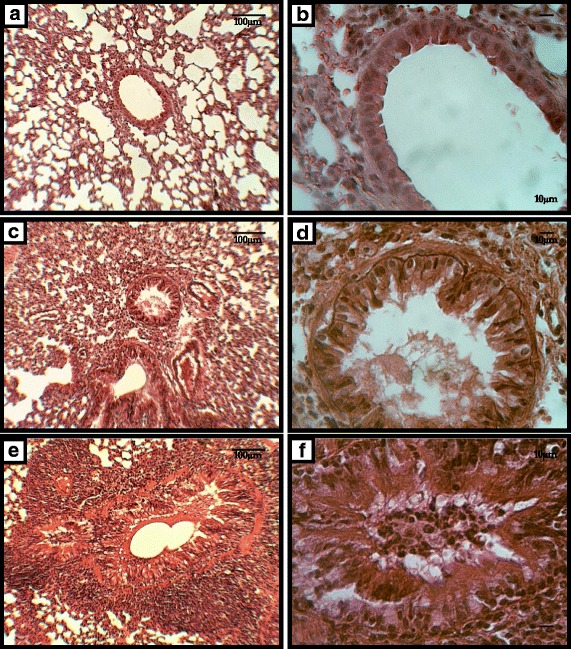
Representative histological features of normal mice lung (**a**) and a normal bronchus (**b**) compared with micrographs of lungs obtained from mice 17 days after the initial challenge with *Aspergillus fumigatus* (**c** & **d**) and of lungs from animals pretreated with cyclophosphamide and exposed to *Aspergillus fumigatus* (**e** & **f**)

Lastly, lung histopathology of animals challenged with *Aspergillus fumigatus* (Fig. 6) showed that the group of animals pretreated with Cy presented significant peribronchiolar and perivascular mononuclear and neutrophilic infiltration, accumulation of intraluminal bronchiolar mucus and serofibrinous exudates. Moreover, there was extensive folding of the airway epithelia, which is a sign of airway smooth muscle contraction and smooth muscle hypertrophy. This inflammatory response was observed predominantly in the peribronchiolar interstitial tissues and often extended into the lamina propria and luminal surface epithelium of the affected airways. We also observed infiltration of inflammatory cells into the alveolar space.

## Discussion

In this paper, we describe that the transient decrease of CD4+, CD25+, Foxp3+ splenic T regulatory cells, induced by Cy, correlates with increased airway inflammation in mice exposed to hay contaminated with *Aspergillus fumigatus*.

The results presented here demonstrate that, in our experimental model, older animals (8 months old) showed a higher percentage of CD4+, CD25+, Foxp3+ cells in the spleen when compared to younger mice (2 months old). Similar findings were reported by Zhao [28], who also compared the capacity of CD4+, CD25+ cells from older and younger mice to inhibit delayed hypersensitivity, mixed lymphocyte reaction or cytokine production. From their results, they concluded that CD4+, CD25+ cells present in the older mice, were less effective in suppressing the above-mentioned assays. These facts may explain why aging produces a progressive decline in immune regulation, and in the case of horses and in the mice of our experimental model [20], why age facilitates the occurrence of airway immune-mediated inflammation. On the other hand, our results indicate that upon stimulation only young mice show a significant increase of CD4+, CD25+, Foxp3+ cells percentage in the spleen, allowing us to propose that aging makes these cells less prone to proliferate or be recruited in response to antigenic challenge.

In addition, we demonstrate that Cy at low doses induces a statistically significant decrease in CD4+, CD25+, Foxp3+ cells in the spleens of mice challenged with *Aspergillus fumigatus*, as described in normal mice by Barbon et al. [22]. This treatment does not affect the relative percentages of other T subpopulations, and this effect has been explained by the presence of lower ATP levels in Treg cells than in conventional T cells. This low ATP content, in turn, attenuates the synthesis of glutathione, which results in a lower capacity for Cy detoxification [28, 29]. Moreover, Lutsiak et al. [30] reported that Cy decreased Treg cell numbers and led to decreased functionality by decreasing GITR and Foxp3 expression, a fact that added to the percentage diminution of Treg and could account for the exaggerated airway immune response observed in animals that received Cy. The fact that a low dose of Cy treatment prior to challenge with *Aspergillus fumigatus* correlated with augmented inflammation and an increase in specific immunoglobulins against the fungus in BALF, may indicate the regulatory role of Treg in this process. In this respect, IgE and IgG1 isotypes are especially important because both have the ability to bind to the high-affinity IgE Fc receptors on mast cells and basophils; therefore, their enhanced production correlates with increased sensitization of the airways and a more aggravated hypersensitivity type I reaction. On the other hand, the IgG2a isotype, which is the only IgG subclass that fixes complement in mice, appears to be responsible for type III hypersensitivity because, upon forming immunocomplexes with the antigen, complement is activated and pro-inflammatory factors such as C3a and C5a are released. Therefore, we may conclude that the increase in immunoglobulins that are responsible for type I and III hypersensitivity, could directly influence the pathogenesis of airway immune-mediated inflammation. As a consequence of antigen-antibody complex formation in the airways, complement activation and the production of other chemoattractants by resident cells in our experimental model mice and in severe equine asthma, neutrophils migrate to the airways, producing an increase of these cells in BALF, which serves as an important diagnostic finding for this disease. Our results show that there is an important increase in BALF neutrophils of mice exposed to *Aspergillus fumigatus*, and this phenomenon is even greater in mice pretreated with low doses of Cy, which indicates that the airway immune-mediated inflammation process induced by the fungus is aggravated by Cy. This conclusion is corroborated by the histopathological study that showed that the highest degree of lung infiltration was found in animals exposed to the antigen and pretreated with Cy before challenge. Finally, considering that the natural thymus-derived, or peripherally induced adaptive Treg cell populations have been associated with the outcome of processes such as autoimmunity, transplantation rejection, cancer and mucosal tolerance [31]; it is not surprising that local airway-induced inflammation correlates with an increase of splenic Treg cells, and a regulatory role of this population as was described here. One of this study’s limitations is the lack of information about specific Treg changes at the site of inflammation (the airways); Treg subpopulations should ideally be evaluated in the lungs and in BALF. However, these results do reveal the systemic effect of Cy on Treg populations, and strongly suggest participation of this cell subset in inflammatory processes. Another limitation of this study is that Cy has been shown to induce lung inflammation at high doses in rats [32]. While in our experience this is not noticeable through the parameters evaluated in this study (histology or BALF relative cellular content, data not shown), this does not exclude the possibility that potential damage induced by Cy may have influenced the inflammatory response against *Aspergillus fumigatus*.

Our study group has previously reported a similar Treg cell subpopulation in horses with exacerbated severe EA [19]. Treg subpopulations may well play a regulatory role in inflammatory processes in different tissues, and our findings here certainly show that inhibition of Treg (through pre-treatment with Cy) coincides with severe airway inflammation after inhalatory challenge. However, translation of these results to the equine species is limited by horses´ individual susceptibility to EA. Multiple factors may affect horses´ susceptibility to inhalatory allergens, including heritable or genetic traits. Familial traits have been proven in said species, and the same has been shown in humans [21, 33]. The murine strain used in this study doesn’t have an increased individual susceptibility to airway inflammation, and as such does not relate specifically to EA-susceptible horses; however, the immunological processes described here are valid across species, and could be applicable to equine susceptible and non-susceptible individuals, albeit the necessary limitations inherent to any research model.

## Conclusion

In this murine model of asthma, the reduced number and function of Treg induced by low doses of Cy and which directly correlates with increased airway inflammation and lung infiltration, indicates that Treg may play a role in the regulation and resolution of severe equine asthma.

## Abbreviations

BALF: Bronchoalveolar Lavage Fluid
Cy: Cyclophosphamide
EA: Equine asthma
OPD: O-phenylenediamine
RK: Rockefeller
Treg: T regulatory cells
